# Musical instrument engagement across the life course and episodic memory in late life: An analysis of 60 years of longitudinal data from the Wisconsin Longitudinal Study

**DOI:** 10.1371/journal.pone.0253053

**Published:** 2021-06-24

**Authors:** Jamie L. Romeiser, Dylan M. Smith, Sean A. P. Clouston

**Affiliations:** 1 Program in Public Health, Department of Family, Population, and Preventive Medicine, Stony Brook University, Stony Brook, NY, United States of America; 2 Department of Anesthesiology, Stony Brook University, Stony Brook, NY, United States of America; University of California, San Francisco, UNITED STATES

## Abstract

**Background:**

As the global burden of dementia increases, the absence of treatment underscores the need for identification of factors that may improve cognitive reserve–the ability to stave off cognitive decline in old age. The beneficial association between musical instrument engagement and episodic memory has been identified in children, young adults, and older adults. Yet, previous studies in musical instrument engagement have rarely examined the potential for adolescence and adulthood exposures to independently improve cognition, nor have they been linked with the rate of memory decline over time in older adults. We investigated whether adolescent musical instrument engagement and continued musical instrument engagement over the adult life course were separately associated with higher episodic memory, as well as rate of decline in a large longitudinal cohort.

**Methods:**

Data were from a prospective cohort of high school graduates from 1957. High school music engagement (HSME) was ascertained through graduate yearbooks and assessed as membership in musical performance groups. A questionnaire was used to assess musical engagement through adulthood (MEA) at ages 35, 55, and 65. The episodic memory score was composed of immediate and delayed recall task scores, and was assessed when participants were aged approximately 65 and 72 years old among 5,718 individuals. Linear mixed models were used to assess the association between music, and memory performance and decline over time.

**Results:**

Of high school graduates who participated in the study, 38.1% played music in high school, and 21.1% played music in adulthood. While musical engagement was more common in those who played in childhood, 40% of those who played continuously as an adult did not play in high school. High HSME (*B* = 0.348, *p* = 0.049) and continuous MEA (*B* = 0.424, *p* = 0.012) were associated with higher memory scores at age 65 after covariate adjustment. When examining memory decline, the benefits of high HSME decreased over time (*B* = -0.435, *p* = 0.048), while the rate of decline did not differ between MEA groups. Exploratory models revealed differential benefits for HSME and immediate recall, and MEA and delayed recall.

**Conclusion:**

This study provides further evidence that musical engagement in childhood or adulthood is associated with non-musical cognitive reserve. These two exposures may act differentially in different domains of episodic memory. Further work is needed to determine the relationship between musicianship and the rate of cognitive decline.

## Introduction

As the world’s population ages, there is an absolute increase in the number of individuals with age-related cognitive diseases such as dementia [1]. Current estimates of the global burden of disease suggest that there are over 43 million individuals living with dementia, and further suggest that this number is growing by 38.2% per year [2]. The absence of a treatment for cognitive decline and dementia underscores the need for identification of factors that may improve “cognitive reserve,” defined as the ability for the brain to continue operating despite increasing levels of age-related pathology, thereby protecting against age-related cognitive impairment [3].

One potentially protective factor against age-related cognitive decline and dementia is musical training [4,5]. Increasing evidence has demonstrated that musical training, or musical instrument engagement, may alter the function and structure of the brain; potentially rendering it more complex and efficient [6–8]. Compared to non-musicians, musicians have demonstrated younger brain-age as measured by structural MRI’s [7]. Years of musical training has shown positive correlations with volume of parahippocampal cortex in older adults [8]. Similarly, musical training is associated with enlargement of the anterior corpus callosum, left temporal lobe, differences in gray matter, and additional macro and microstructural brain changes have been identified in those with musical training compared to those without musical training [9–11].

These structural differences are believed to indicate functional reorganization, which may be associated with benefits in certain cognitive functions for musically-trained individuals [12]. Studies in children have revealed cognitive advantages amongst those who have received musical training compared to those that did not [13–16]. For example, children in a 15-month musical intervention group saw greater improvements in overall cognitive performance compared to control groups of drama lessons and no intervention [15]. Further, children in a two-year intensive musical instrument training program manifested broad cognitive benefits including working memory, attention, and processing speed compared to a musical listening and theory program control [16].

These cognitive advantages among musically-trained individuals may continue into older age [17–23]. A recent meta-analysis analyzed 9 correlative studies and 4 experimental studies in musicians versus non-musicians age 59 and over [20]. Most of the studies included defined musicianship as having early (adolescent) and long-term (current) training. Musicianship was beneficially associated with a wide array of cognitive functioning areas such as visuospatial ability, verbal working memory, and auditory perception. Potentially more interesting, adults who engaged in musical training at a young age exhibited sharper neural processing and higher cognitive reserve later in life, irrespective of current musical engagement levels [24,25].

Yet, while cross-sectional research is suggestive of improvements to cognitive function in adults, it remains unclear whether musicianship is associated with the rate of cognitive decline in later life. Randomized trials could examine musical training interventions and cognitive decline in older aged participants, but these types of studies are not common [20], and follow up is usually brief. One trial in participants aged 60–85 found that six months of piano lessons provided some benefit in working memory compared to the no interventional group, though benefits waned after lessons stopped, and it was unknown if there were other lasting effects beyond the 9-month study period [26].

While longitudinal cohort studies can rarely tease out direct causal relationships, they provide a unique perspective on aging over longer periods of time in the same group of individuals. One longitudinal study showed that early-life musical engagement (>4 years before the age of 18) was related to both improved episodic memory and longer time to diagnosis of mild cognitive impairment, but not with rate of cognitive decline over 5 years [25]. A second longitudinal study investigated associations between leisure activities and risk of dementia in adults over 75 years of age [27], finding that frequent musical instrument engagement (several days or more per week) at baseline was associated with lower risk of dementia, while frequent leisure activity participation reduced the rate of cognitive decline over 5 years.

Musical instrument engagement in adulthood is less common amongst individuals who were never trained as children [28]. It, therefore, remains unclear whether instrument engagement in adulthood plays a role in providing cognitive reserve above and beyond childhood exposures. In this longitudinal study, we focus on separate effects of adolescent and adult musical instrument engagement over the life course, and episodic memory. Episodic memory is common in studies of cognitive aging, and is a hallmark symptom used in identifying individuals with Alzheimer’s disease and other related dementias [29–31]. Episodic memory, and verbal components of episodic memory, have demonstrated associations with musical instrument play across many studies. Children who played an instrument for at least one year demonstrated higher verbal immediate and delayed recall in memory tasks compared to those who did not [14]. In college students, verbal memory scores were higher amongst those who received musical training in adolescence compared to those with no training [17,32]. A meta-analysis in young adults [4] and older adults [20] confirmed similar benefits in verbal learning and working memory associated with musicianship.

While there is a strong associative base of literature on musical training and episodic memory, there are limited data looking at the separate effects of playing music in the younger years as well as continued play throughout the life course, and the association with episodic memory in older age. Furthermore, little is known about whether separate musical training exposures are associated only with higher lifetime memory performance, or if it may also help to slow the rate of decline. To our knowledge, there are no longitudinal studies investigating the relationship between music training and memory decline using distinct adolescent and adult musical instrument engagement assessments. Thus, using data from an observational longitudinal cohort study, we hypothesized that 1) adolescent musical instrument engagement and continued musical instrument engagement over the life course are separately associated with higher episodic memory in later life; and 2) adolescent and continued musical engagement over the life course are protective against memory decline.

## Methods

### Participants

We conducted secondary data analyses using data from the Wisconsin Longitudinal Study (WLS), funded by the National Institute on Aging [33]. The WLS is a life course study of 10,317 randomly-selected men and women who graduated from Wisconsin (USA) high schools in 1957. Approximately one third of all Wisconsin state graduates in 1957 were selected for this long-term follow-up study. Survey data on the graduates were collected from both graduates and their parents, with collection waves occurring in 1957, 1964, 1975, 1993, 2004, and 2011.

### Ethics

All data are deidentified and publicly available for download (https://www.ssc.wisc.edu/wlsresearch/data/), and therefore exempt from Institutional Review Board approval.

### Outcome—Episodic memory

In the WLS follow up survey years 2004 and 2011, multiple domains of verbal cognition were assessed during telephone or in-person interviews using the Wechsler Adult Intelligence Scale–Revised and other cognition measurements. The episodic memory score was composed of two components: immediate and delayed recall task scores. These tasks were specifically developed to be administered to participants over the telephone, have shown a high sensitivity and specificity to diagnose Alzheimer’s disease, and have a high test-retest reliability in older populations [31]. Details on administration of these tasks may be found in the Wisconsin Longitudinal Study instrument handbook, available on the site listed above. Briefly, for verbal learning, interviewers read a list of ten high-frequency words, one every two seconds. Respondents were given up to two minutes to immediately and correctly recall as many words as possible from this list. Interviewers then distracted the respondents with other questions including information on insurance, pensions, and retirement attitudes for ~12 min to avoid interviewee rehearsal of the list. Then, long-term memory was assessed by asking participants to verbally recall the same list from the immediate recall task. The number of words correctly recalled for both the immediate and delayed task were recorded. The primary outcome of episodic memory was defined as the summation of these measures. In both the general aging literature as well as music specific cognition literature, these tasks are often combined to form one measure of episodic memory [14,25,34–36]. However, we also performed two exploratory analyses that evaluated separate models for the immediate recall and delayed recall tasks.

We are not aware of any norms available with which to objectively determine levels and types of cognitive impairment in this study. However, in an effort to determine clinical relevance of the impact musical engagement may have on memory, a separate sensitivity analysis was performed. Using a common guideline from prior mild cognitive impairment (MCI) studies [37], we operationalized possible MCI as a performance of 1.5 standard deviations below the mean episodic memory score in 2004. This analysis was exploratory.

### Primary predictors—Measures of musical instrument engagement

Adolescent engagement in musical performance groups in high school served as the primary measure of early musical involvement. WLS researchers obtained yearbooks for graduates included in original study. All student activities listed within the yearbook, including the number of musical performance groups, were coded for each graduate. The musical performance variable summarized the total number of orchestra, band, chorus, and smaller musical ensemble activities in the senior year. As noted in White-Schwoch [24], high school participation in a musical group generally corresponds with 4–14 years of musical training. Further, students involved in more than two musical groups in high school are likely to have invested a greater length of time and intensity in musical training through the earlier years of their life. The distribution of this variable was also skewed and provided a natural cutoff, with notably fewer students participating in 3, 4, 5 or 6+ musical groups. Therefore, *High School musical engagement (HSME)* was categorized into three groups: *no musical participation* (0 musical performance groups), *moderate* participation (1–2 groups), and *high* participation (3+ groups).

**Fig 1:**
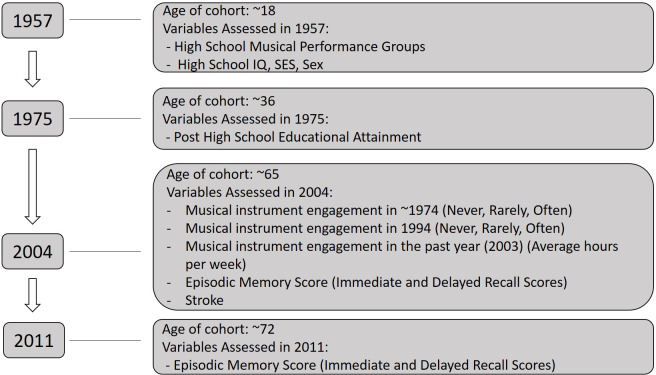
Flow diagram of variable assessments. Data were collected at multiple timepoints from 1957 when participants were approximately 18 years of age, through 2011 when participants were approximately 72 years of age.

Continued musical engagement was assessed through several questions in 2004 and 2011 follow up surveys. In 2004, graduates were asked: 1) the duration of time spent per month playing a musical instrument in the past year (2003); 2) how often they played an instrument 10 years prior (1994, categorized as never, sometimes, often); and 3) how often they played an instrument at age 35 years (~1974, categorized as never, sometimes, often). Data collection points are outlined in **Fig 1**.

Musical engagement in adulthood (MEA) was operationalized as a composite variable, with *continuous play*, *intermittent play*, and *never play* defined as the following: continuous play is defined as instrument engagement all follow-up time points (e.g., any musical instrument engagement in 1974, 1994, and 2004); intermittent play is defined as those who played at one point or another through time, but did not play continuously; and never play was defined as those who never engaged in musical instrument play at any of the follow up time points.

Because the primary outcome, episodic memory, was first assessed in 2004, we decided not to include musical practice from the 2011 assessment to avoid reverse causality (i.e., an association between less musical practice in 2011 and lower memory scores that is actually due to a decline in memory after 2004). As a sensitivity analysis, we investigated including musical practice in 2011 as a separate variable. However, we found play in 2011 to be highly correlated with the MEA variable. This indicated that musicianship in adulthood was relatively stable. Indeed, very few individuals began to play in 2011 who had never played previously as an adult. There was a slightly higher percentage of continuous play participants who stopped playing in 2011, but removing these individuals from the analysis made no substantive difference in our results.

### Covariates

Based on this previous literature, covariates were identified and included in all adjusted models. High School IQ score was defined as the centile rank based on National test takers for Henmon-Nelson score. This variable was standardized in the adjusted models for ease of interpretation. Educational attainment was categorized as high school graduate, Associate’s degree, Bachelor’s degree, or completed/completing a graduate or professional degree. Sex and any previous stroke were included. Race and ethnicity were not included, as there was little diversity in this Wisconsin-based sample. Age was also not included, as all participants were approximately the same age.

Using components described in Greenfield and Moorman [38], a global childhood SES variable was constructed using standardized measures for father’s 1957 job income, father’s education, parental income, and 1950 National Opinion Research Center (NORC) prestige score for father’s occupation 1957. All individual standardized measures were then averaged to form a composite, global measure of childhood SES.

### Statistical analysis

Participants were included for analysis if they performed the verbal learning and memory tasks during the 2004 follow-up assessment or the 2011 follow-up assessment (n = 5,718). Univariate analyses compared demographic covariates and later life cognition outcomes across three levels of high school music participation (none, moderate, high) using chi-square and ANOVA tests. Linear mixed models were used to assess the association between music and memory scores. Scores at baseline (i.e., episodic memory at age 65) were allowed to vary across participants (i.e., random intercept). Random slopes were examined but not included because the resulting variance component for the slopes was both small and insignificant. Memory decline estimates by musical engagement groups are represented by the interaction of time and the music engagement variables. High school music engagement (HSME) groups and continued music engagement after high school (MEA) were first modeled separately (Model 1 and Model 2), and then together (Model 3). An interaction term between HSME and MEA was found to be insignificant for all subgroups and therefore removed from the model. Additional covariates were controlled for in Model 4. In an exploratory analysis, the episodic memory outcome was divided into its two separate components: immediate recall (Model 5) and delayed recall (Model 6). Covariance matrices were specified as unstructured. Collinearity was examined for all fixed model parameters using both correlation matrixes and simple linear regression with variance inflation factors estimated. All rhos were less than 0.3 and all VIF estimates were below 1.4, indicating low risk of multicollinearity. All analyses were performed using SAS 9.4© Cary, N.C.

## Results

Descriptive data are presented in **Table 1**. Respondents were evenly split by sex (54% female). Most respondents did not continue their education (74% high school graduates). Sixty-two percent were not involved in musical performance groups in high school, 29% were involved in 1–2 musical performance groups, and 9% were involved in 3 or more musical performance groups. After high school, 55% did not engage in musical instrument play, 14% played intermittently, 8% played continuously, and 23% could not be categorized or were unknown. Forty percent of those who played continuously as an adult did not play in high school (172/428). Average memory scores in 2004 and 2011 were 10.25 (SD 3.62) and 8.91 (SD 2.92), respectively.

**Table 1:** Descriptive statistics, demographics by high school music engagement.

Demographics	Total	None (0 HSME Groups)	Moderate (1–2 HSME Groups)	High (3+HSME Groups)	*P* -value
**Total—n (%)**	5718 (100%)	3541 (61.93%)	1687 (29.50%)	490 (8.57%)	
**Sex** (Female)–n (%)	3082 (53.90%)	1564 (44.17%)	1150 (68.17%)	368 (75.10%)	<0.01
**Education–**n (%)					
High School	4210 (73.63%)	2659 (75.09%)	1232 (73.03%)	319 (65.10%)	<0.01
Associates	104 (1.82%)	65 (1.84%)	27 (1.60%)	12 (2.45%)	
Bachelors	921 (16.11%)	524 (14.80%)	296 (17.55%)	101 (20.61%)	
Graduate	483 (8.45%)	293 (8.27%)	132 (7.82%)	58 (11.84%)	
**Music Engagement in Adulthood (MEA)–**n (%)					
Never Played	3169 (55.42%)	2106 (79.17%)	882 (67.07%)	181 (45.14%)	<0.01
Played Intermittently	779 (13.62%)	382 (14.36%)	280 (21.29%)	117 (29.18%)	
Played Continuously	428 (7.49%)	172 (6.47%	153 (11.63%)	103 (25.69%)	
Unknown	1342 (23.47%)				
**High School IQ Centile–**mean (SD)	60.53 (25.72)	59.06 (26.00)	61.50 (25.31)	67.76 (23.62)	<0.01
**Global Childhood SES—**standardized mean (SD)	0.01 (0.83)	0.01 (0.843)	0.01 (0.81)	0.08 (0.83)	0.16
**Stroke** *(Any time prior to 2004 interview)–*n (%)	180 (3.15%)	113 (3.36%)	55 (3.40%)	12 (2.53%)	0.62
**Episodic Memory Score 2004 –**mean (SD)	10.25 (3.62)	9.98 (3.56)	10.57 (3.61)	11.09 (3.83)	<0.01
**Episodic Memory Score 2011—**mean (SD)	8.91 (2.92)	8.73 (2.88)	9.09 (2.97)	9.53 (3.00)	<0.01

Differences in the distribution of demographic variables were described by high school music engagement groups.

HSME = high school music engagement; IQ = intelligence quotient; SD = standard deviation; SES = socioeconomic status. P-values were calculated using chi-squares for categorical variables and ANOVA tests for continuous variables.

Sex, education, post high school musical instrument engagement, and high school IQ all significantly differed between the high school musicianship groups (p<0.01, **Table 1**). A greater proportion of females participated in musical groups. Those who were highly involved in high school music tended to acquire more post-high school education, and a greater proportion of them continued their musicianship. As high school musicianship increased, so did 2004 and 2011 episodic memory scores (p <0.01).

High school and continued musical instrument engagement were first modeled separately (Model 1 and Model 2), and then together (Model 3) to parse out possible adolescent and adult associations with memory and decline (**Table 2**). In Model 1, moderate and high levels of high school musical engagement (HSME) were significantly associated with higher memory scores at baseline (i.e., memory at age 65) compared to no HSME. In fact, the size of the coefficient for the high level of HSME was almost twice that of the moderate HSME group. Increased rate of decline over time for both groups was marginally significant, indicating a possible trend in regression back to the mean. Similar results and coefficients were found for music engagement as an adult. In Model 2, both intermittent and continuous MEA were significantly associated with higher memory scores at baseline compared to no MEA. However, neither were associated with a decline in memory scores. In Model 3, most of the effects demonstrated a small attenuation, but significant effects from the univariate models remained significant in the combined model. Compared to no high school music, both moderate and high HSME showed higher average memory scores at age 65 (*B* = 0.498, *p* < 0.001, *B* = 0.958, *p* < 0.001). Both groups showed a steeper rate of decline compared to no HSME, but results were moderately significant (*B* = -0.235, *p* = 0.088, *B* = -0.041, *p* = 0.058). Musical engagement in adulthood showed a similar dose response relationship at baseline (intermittent MEA *B* = 0.551, *p* < 0.001; continuous MEA *B* = 0.921, *p* < 0.001), but neither showed a significant effect over time compared to the no adult musicianship group.

**Table 2:** Adolescent and adult musical engagement and memory decline.

Outcome: Episodic Memory	MODEL 1: HSME*TIME			MODEL 2: MEA*TIME			MODEL 3: Music*TIME		
Effect	Estimate	STE	*p—*Value	Estimate	STE	*p—*Value	Estimate	STE	*p—*Value
Intercept	9.950	0.058	< .0001	10.118	0.060	< .0001	9.924	0.069	<0.001
Time	**-1.240**	**0.070**	**< .0001**	**-1.273**	**0.072**	**< .0001**	**-1.183**	**0.084**	**<0.001**
HSME (None)	*Reference*						*Reference*		
HSME (Moderate)	**0.583**	**0.102**	**< .0001**				**0.498**	**0.113**	**<0.001**
HSME (High)	**1.112**	**0.164**	**< .0001**				**0.958**	**0.183**	**<0.001**
HSME (Moderate * Time)	-0.203	0.123	0.100				-0.235	0.138	0.088
HSME (High *Time)	-0.358	0.196	0.068				-0.414	0.219	0.058
MEA (None)				*Reference*			*Reference*		
MEA (Intermittently)				**0.682**	**0.134**	**< .0001**	**0.551**	**0.135**	**<0.001**
MEA (Continuously)				**1.139**	**0.171**	**< .0001**	**0.921**	**0.175**	**<0.001**
MEA * Time (Intermittently)				-0.167	0.161	0.299	-0.109	0.163	0.502
MEA * Time (Continuous)				-0.249	0.206	0.225	-0.153	0.210	0.468
**Covariance Parameters**	**Estimate**	**STE**	**p**	**Estimate**	**STE**	**p**	**Estimate**	**STE**	**p**
Between Subjects	0.008	0.182	< .0001	3.808	0.195	< .0001	3.749	0.194	<0.001
Residual	6.927	0.155	< .0001	6.818	0.168	< .0001	6.815	0.168	<0.001

Results from 3 simple linear mixed models are presented, including HSME * Time (Model 1), MEA * Time (Model 2), and all music variables by time (Model 3).

STE = Standard error; HSME = High School Music Engagement; MEA = Music Engagement as an Adult. Bolded model parameters indicate significance.

**Fig 2:**
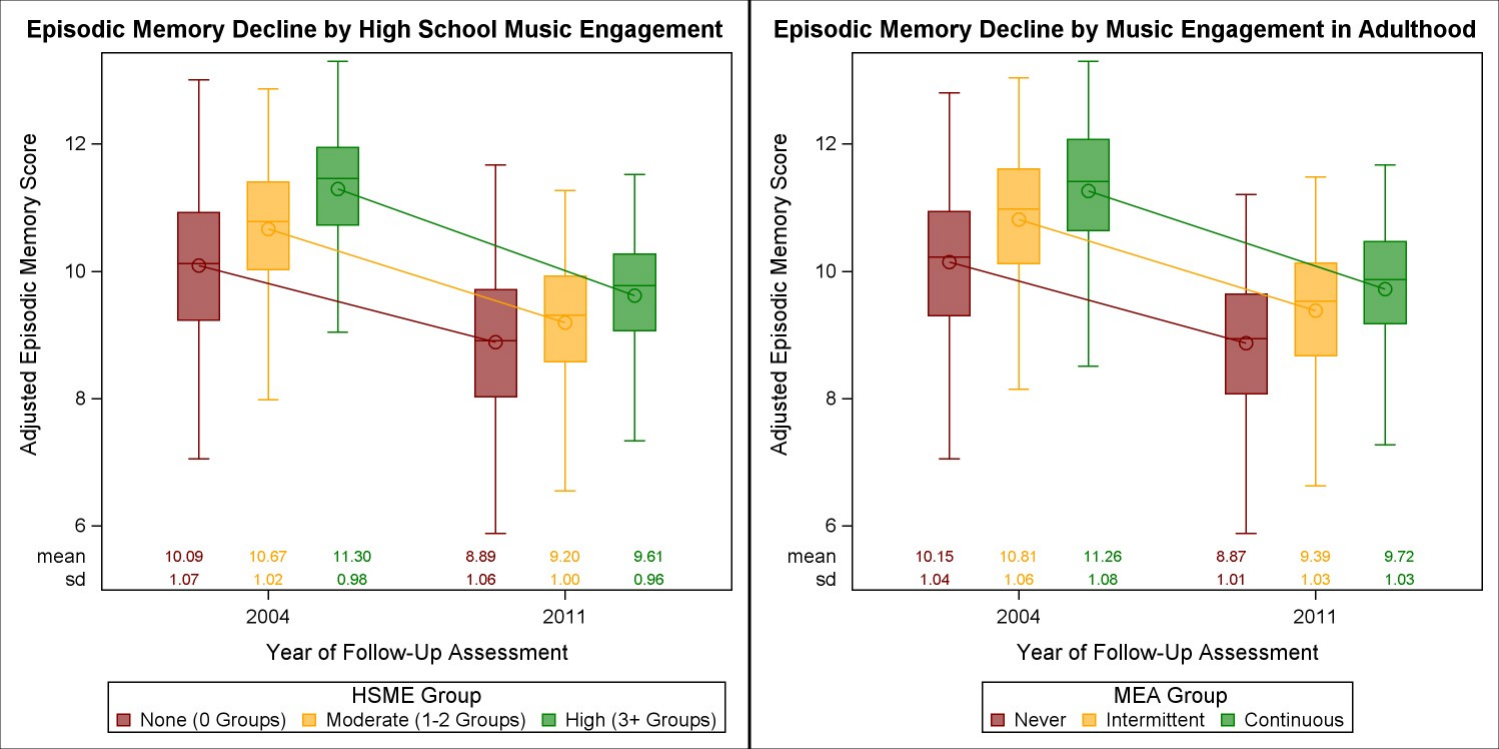
Change in adjusted episodic memory scores for musical engagement groups. (**A**) Adjusted predicted memory scores for high school musical engagement (HSME) groups are plotted at both time points. There were significant differences in the adjusted scores between High HSME group and the No HSME group at baseline (*B* = 0.348, *p* = 0.049). Slopes between these groups also significantly differed, indicating a steeper rate of decline for the High HSME group compared to the No HSME group (*B* = -0.435, *p* = 0.049). **(B)** Adjusted predicted memory scores for musical engagement in adulthood (MEA) groups are plotted at both time points. There were significant differences in the adjusted scores between the Continuous MEA group and the No MEA group at baseline (*B* = 0.424, *p* = 0.012). Rate of decline (slopes) did not differ between the groups.

Once adjusted for additional covariates in Model 4 (**Table 3**), both high HSME and continuous MEA again attenuated but remained significant positive predictors of memory at baseline. High HSME continued to show a significantly steeper cognitive decline (*B* = -0.435, *p* = 0.048 [**Fig 2A**]). The rate of cognitive decline did not differ between continued MEA groups (**Fig 2B**).

**Table 3:** Adjusted models to predict episodic memory, immediate recall and delayed recall scores.

Effect	Model 4: Episodic Memory			Model 5: Immediate Recall Only			Model 6: Delayed Recall Only		
	Estimate	STE	*p—Value*	Estimate	STE	*p—Value*	Estimate	STE	*p—Value*
Intercept	9.118	0.082	< .0001	5.676	0.039	< .0001	3.478	0.050	< .0001
Time	**-1.177**	**0.085**	**< .0001**	**-0.598**	**0.043**	**< .0001**	**-0.568**	**0.051**	**< .0001**
HSME (None)	*Reference*			*Reference*			*Reference*		
HSME (Moderate)	0.087	0.111	0.431	**0.101**	**0.054**	**0.061**	-0.004	0.067	0.955
HSME (High)	**0.348**	**0.177**	**0.049**	**0.338**	**0.086**	**< .0001**	0.047	0.108	0.663
HSME (Moderate * Time)	-0.256	0.139	0.065	**-0.136**	**0.071**	**0.054**	-0.130	0.084	0.123
HSME (High *Time)	**-0.435**	**0.220**	**0.048**	**-0.354**	**0.112**	**0.002**	-0.121	0.133	0.360
MEA (None)	*Reference*			*Reference*			*Reference*		
MEA (Intermittently)	0.227	0.130	0.081	-0.009	0.063	0.885	**0.207**	**0.080**	**0.009**
MEA (Continuously)	**0.424**	**0.169**	**0.012**	0.132	0.082	0.107	**0.278**	**0.103**	**0.007**
MEA * Time (Intermittently)	-0.091	0.164	0.581	0.018	0.083	0.825	-0.077	0.099	0.435
MEA * Time (Continuous)	-0.168	0.212	0.428	-0.019	0.107	0.859	-0.132	0.128	0.303
Global Childhood SES (Standardized)	0.093	0.051	0.066	**0.049**	**0.024**	**0.044**	0.047	0.031	0.133
Sex (Female vs. Male)	**1.689**	**0.085**	**< .0001**	**0.734**	**0.041**	**< .0001**	**0.952**	**0.052**	**< .0001**
Education (High school)	*Reference*			*Reference*			*Reference*		
Education (Associates)	0.208	0.292	0.478	0.050	0.140	0.722	0.173	0.179	0.335
Education (Bachelors)	**0.433**	**0.114**	**0.0001**	**0.207**	**0.054**	**0.0001**	**0.229**	**0.070**	**0.001**
Education (Graduate)	**0.814**	**0.151**	**< .0001**	**0.422**	**0.072**	**< .0001**	**0.400**	**0.093**	**< .0001**
High School IQ (Centile) (Standardized)	**0.486**	**0.047**	**< .0001**	**0.260**	**0.022**	**< .0001**	**0.227**	**0.029**	**< .0001**
Stroke	**-1.071**	**0.241**	**< .0001**	**-0.543**	**0.115**	**< .0001**	**-0.530**	**0.148**	**0.000**
**Covariance Parameters**	**Estimate**	**STE**	***p—Value***	**Estimate**	**STE**	***p—Value***	**Estimate**	**STE**	***p—Value***
Between Subjects	2.709	0.175	<0.001	0.500	0.041	< .0001	1.069	0.065	< .0001
Residual	6.821	0.169	<0.001	1.794	0.044	< .0001	2.480	0.061	< .0001

Adjusted linear mixed models for the primary outcome (episodic memory) as well as immediate and delayed recall are presented.

STE = Standard Error; HSME = high school music engagement; MEA = music engagement in adulthood; IQ = intelligence quotient; SES = socioeconomic status. Bolded model parameters indicate significance at the *p* ≤ 0.05 level.

Overall, memory scores for participants decreased over time approximately 1.2 points on average (*B* = -1.177, *p* < 0.001*)*. Global childhood SES, sex, education, high-school IQ, and stroke were also significant or marginally significant. After controlling for all other model covariates, memory scores for females were approximately 1.7 points higher on average compared to males, and those who had a stroke had approximately 1.1 points lower on average compared to those who did not. For every standard deviation above the mean high school IQ, memory scores increased approximately 0.49 points.

Episodic memory was dichotomized into a possible indication of MCI as a sensitivity analysis, but presence in the study population was low (4.2% in 2004, and 6.7% in 2011). In the adjusted model, most of the results for the covariates remained the same. High HSME was protective but became marginally significant (OR 0.39, p = 0.07), and MEA became insignificant (model results not shown).

Episodic memory was comprised of both immediate and delayed recall tasks. However, we performed two additional exploratory models to examine these tasks separately (Model 5 and Model 6, **Table 3**). As seen in previous literature, most of the covariate effects were split relatively evenly between the immediate and delayed recall models. For example, the coefficient of stroke in the immediate recall model was *B* = -0.543; in delayed recall model, stroke *B* = -0.530. Interestingly, the HSME and MEA did not mirror this even split between the two tasks. Rather, moderate and high HSME were both significant positive predictors of immediate recall, whereas MEA was not. Both moderate and high HSME also saw significant decline over time compared to no HSME. Conversely, moderate and high MEA were both significant positive predictors of delayed recall, whereas HSME was not. Similar to the full memory model (Model 4), there was no significant association between MEA subgroups and decline over time.

## Discussion

Limited information is currently available about the extent to which musical engagement might offset or delay the onset of declines in episodic memory later in life. Using a longitudinal cohort of Wisconsin high school graduates first studied in 1957, we examined the association between musical instrument engagement during high school or thereafter and episodic memory and decline in late life. Consistent with previous research, we found a significant association between musical instrument engagement and memory. Separately, both high early-life involvement and continued instrument engagement in adulthood were significantly associated with higher cognition scores at baseline, after adjusting for covariates. When examining rate of decline, the benefits of high HSME appeared to regress back to the mean. Conversely, no significant differences were found in the rate of decline and different levels of MEA.

Our study results are consistent with previous literature demonstrating a lingering baseline cognitive benefit in older age that was associated with adolescent musical training [24,25]. Even though benefit declined over time in our study for the high HSME group, 2011 episodic memory scores for the high HSME group were still significantly higher compared to no HSME group. Our continued MEA results were similar to findings seen in an older adult study [27], which found that instrument engagement at an older age was associated with a decreased risk of dementia.

This study sought to examine an activity that might influence the brain’s cognitive capacity over the life course. Specifically, we hypothesized that musical training had the potential to benefit memory function across the life course, thereby improving cognitive reserve into late life. Notably, while we expected that childhood would be a sensitive period in terms of the effects of exposure to musical training, we are relatively unique in finding that musical training in childhood *or in adulthood* were independently associated with higher cognition in old age. Our study, therefore, differs from many studies in the cognitive reserve literature that focus either on early life or on late-life activities without consideration of prior exposures. The benefits of musical instrument engagement seem to build throughout the life course, and these associations were similar in size in late life as they were in early life. The effect of MEA was not moderated by HSME, and these two variables were not highly correlated according to variance inflation factors. Our models show positive effects for both variables, suggesting that there may not be a critical or sensitive period limiting the impact of early and late-life exposures to musical training.

It’s possible that high levels of childhood engagement may even provide a clinically meaningful benefit in cognitive reserve. In a sensitivity analysis, high HSME was marginally protective against possible MCI after covariate adjustment. This analysis was exploratory; therefore, future research may seek to determine whether cognitive declines identified in this study are consistent with early cognitive decline and the extent to which HMSE and MEA might result in decreased risk of mild cognitive impairment or dementia.

Notably, early and adult engagement may act differentially within the episodic memory domain. Episodic memory encapsulates the ability to learn a specific bit of information and recall both doing it and the content of the task at a later time. This domain of cognitive function has been instrumentalized in different ways, but commonly as a combination of immediate and delayed recall tasks [14,25,30,34,35]. It was not surprising, therefore, when most of the covariates behaved similarly after episodic memory was split into immediate and delayed recall exploratory models. However, it was unexpected that the music variables did not demonstrate the same pattern. After controlling for covariates including adult musical engagement, both moderate and high HSME were positively associated with immediate recall but not with delayed recall. Conversely, after controlling for covariates including adolescent musical play, both moderate and high MEA were positively associated with delayed recall but not with immediate recall.

In all adjusted models, MEA followed the pattern of preserved-differentiation rather than differential-preservation [28,39]. That is, the difference in memory scores seen at age 65 between the high music group and no music group did not grow over time (i.e., no positive significant difference in slopes). It is possible that while instrument engagement throughout the adulthood appears to provide an enhancer effect by building reserve/scaffolding, it may not be protective in mitigating age-related decline in regards to episodic memory. While the high HSME group also demonstrated higher episodic memory scores at both time points compared to the no HSME group, they experienced a significantly steeper decline in memory score from 2004 to 2011. A well-described theoretical explanation for this is that cognitive reserve which is built from beneficial life experiences can act as a moderator between the advancement of neuropathology and onset of functional decline for only so long [40]; yet, once neuropathology overwhelms reserves, those individuals with higher cognitive reserve often see steeper cognitive declines due to the severe nature of the neuropathology [40].

### Our study has several strengths

This is a longitudinal cohort study that assess both the predictors and outcomes at multiple time points. Both childhood and adult musical engagement could be assessed separately in the context of both cognition and cognitive decline. Many music training and cognition studies are plagued by small sample sizes, matching on minimal factors. This is one of the largest studies to date to examine this association, with the ability to control for multiple covariates that are known to affect the outcome.

### Our study also has limitations

Because it is more common to begin an instrument when you are young and quit after several years than it is begin an instrument late in childhood education, we may have inadvertently included individuals into our “no HSME” group who may have had childhood exposure but quit prior to their senior year; this would ultimately bias our HSME effects toward the null. In terms of MEA, we relied on broad and subjective measures of musical engagement, in contrast to many musical training and cognition studies where the musician group is defined under strict professional terms. It could be argued that by using a broader definition of musician that includes less intense practice, we might not necessarily be isolating the effects of music, but rather also capturing a greater propensity to lead more active lives in other ways that increase cognition. While were able to control for adolescent background characteristics that are likely to be associated with maintaining a musical training lifestyle (i.e., higher adolescent IQ and SES), we were not able to control for personality traits, musical aptitude, engagement in intellectual games, physical activity, or other lifestyle activities that may be associated with both musicianship [41] and higher cognitive performance later in life [42,43]. Therefore, the present design does not allow for the disentanglement of the causal role of musical training.

The literature on general lifestyle activity profiles between musicians and non-musicians is mixed. General lifestyle activity profiles may differ in younger adult musicians and non-musicians [44], but perhaps not between older musicians (with >10 years of experience) and non-musicians [22]. Findings on personality trait profile differences between musicians and non-musicians or time trained as a musician is also mixed, depending on the age of the cohort assessed [41,45]. Nonetheless, musical activity has demonstrated significant associated effects with cognition beyond those attributed to other beneficial traits such as physical activity [27,29] bilingualism [25], and personality traits [45].

Active musical engagement in 2011 was highly associated MEA, which induced collinearity issues and was therefore not included as a separate predictor. Musical play in adulthood appeared to be relatively stable for those who never played and those who were always engaged, but we were not able to separately untangle these effects. Prior studies have demonstrated positive associations between current/active musical practice and executive functioning [26,46]. Yet, we did not find large differences in the risk of stroke, the most reliably measured cause of declines in executive functioning that are independent of memory declines, potentially suggesting that associations with executive functioning could replicate those shown here. While the current investigation did not examine these mechanisms, it is possible that current musical practice may additionally improve memory performance by strengthening executive function in older adults [46].

As with all prospective studies, we cannot isolate a single causal factor and rule out the possibility that musical instrument engagement itself is influenced by an unobserved neuropathology. Finally, due to the homogeneity of the cohort, our results are not generalizable across different races.

## Conclusions

This study provides further support to the growing body of evidence suggesting that musical engagement is associated with non-musical cognitive reserve and may delay the onset of clinically-meaningful cognitive impairments later in life. Critically, the late-life benefits can be seen from musical engagement irrespective of the timing of engagement. Therefore, musical instrument engagement in adolescence or adulthood may help to improve cognitive domains such as episodic memory, but may not reduce the rate of decline.
